# Characterization of Mechanical and Bactericidal Properties of Cement Mortars Containing Waste Glass Aggregate and Nanomaterials

**DOI:** 10.3390/ma9080701

**Published:** 2016-08-18

**Authors:** Pawel Sikora, Adrian Augustyniak, Krzysztof Cendrowski, Elzbieta Horszczaruk, Teresa Rucinska, Pawel Nawrotek, Ewa Mijowska

**Affiliations:** 1 Faculty of Civil Engineering and Architecture, West Pomeranian University of Technology, Al. Piastow 50, Szczecin 71-311, Poland; elzbieta.horszczaruk@zut.edu.pl (E.H.); trucinska@zut.edu.pl (T.R.); 2 Faculty of Biotechnology and Animal Husbandry, West Pomeranian University of Technology, Doktora Judyma st. 26, Szczecin 71-466, Poland; adrian.augustyniak@zut.edu.pl (A.A); pawel.nawrotek@zut.edu.pl (P.N.); 3 Faculty of Chemical Technology and Engineering, Institute of Chemical and Environment Engineering, West Pomeranian University of Technology, Al. Piastow 45, Szczecin 71-311, Poland; kcendrowski@zut.edu.pl (K.C.); ewa.mijowska@zut.edu.pl (E.M.)

**Keywords:** waste glass, cement mortar, recycling, nanosilica, titanium dioxide

## Abstract

The recycling of waste glass is a major problem for municipalities worldwide. The problem concerns especially colored waste glass which, due to its low recycling rate as result of high level of impurity, has mostly been dumped into landfills. In recent years, a new use was found for it: instead of creating waste, it can be recycled as an additive in building materials. The aim of the study was to evaluate the possibility of manufacturing sustainable and self-cleaning cement mortars with use of commercially available nanomaterials and brown soda-lime waste glass. Mechanical and bactericidal properties of cement mortars containing brown soda-lime waste glass and commercially available nanomaterials (amorphous nanosilica and cement containing nanocrystalline titanium dioxide) were analyzed in terms of waste glass content and the effectiveness of nanomaterials. Quartz sand is replaced with brown waste glass at ratios of 25%, 50%, 75% and 100% by weight. Study has shown that waste glass can act as a successful replacement for sand (up to 100%) to produce cement mortars while nanosilica is incorporated. Additionally, a positive effect of waste glass aggregate for bactericidal properties of cement mortars was observed.

## 1. Introduction

Concerns related with disposal of generated wastes have increased tremendously in the last two decades. Awareness of the environment and solid waste management gathered major concern throughout the world. Material that especially aroused the interest of many researchers, due to low recycle rate and high disposal costs, is waste glass (WG). The construction industry (especially the cement and concrete industry) found a useful solution for the environmental impact of waste glass. The physical properties of glass and its chemical composition, similar to that of sand and cement, make this material very appealing. It is able to provide an environmentally friendly solution for the glass and cement industries. Theoretically, glass could be recycled completely and infinitely without losing any of its chemical and physical properties [1]. However, broken, mixed colored and diverse origin of waste glass, make the recycling process impractical and highly expensive. Different chemical compositions, impurities and contaminants of recycled glass can highly affect the properties of the produced new glass. Therefore, there is a need to develop markets for mixed waste glass. The use of glass as cement concrete aggregates in last few decades has again come under investigation. Application of waste glass both, as an aggregate or glass powder seems to be a very interesting approach to create ecological composites but there are still some obstacles that need to be resolved.

Extensive studies held by researchers have shown that the optimum replacement of sand with waste glass without deteriorating mechanical properties varies from 10% up to 30% [2,3,4,5,6]. In addition, the experimental studies showed that similar or higher flexural and tensile strength can be obtained in samples containing up to 25% and 20% of sand replacement respectively. Differences in obtained results are mostly due to aggregate sample size and its size distribution [7].

Based on the previous research presented by other researchers, it can be concluded that application of waste glass as an aggregate to cementitious composites has a very promising effect, especially when finer aggregate is used (due to more proper and more spherical geometry of waste glass) [7]. Besides, as reported by Shi and Zheng [8], very fine particles due to high amount of silica SiO_2_ can exhibit additionally pozzolanic activity [5,9,10]. Unfortunately, presence of waste glass in cementitious composite provides the possibility of its expansion and cracking. There is still no agreement regarding the alkali-silica reaction (ASR) in mortars and concrete containing waste glass aggregate. The amorphous silica can be dissolved in glass under alkaline conditions and form ASR gel which absorbs water and subsequently expand leading to tensile stresses. When tensile stress exceeds the tensile strength of the material, cracks will occur. As a result of cracking, corrosive alkali will come into contact with internal glass structure more easily, which will result in ASR acceleration, widening of the cracks, and debilitation in the quality of cement-based composite [11]. Occurrence of ASR is mainly related to the amount, particle size, and color of waste glass [8,12]. Moreover, type of glass (its origin) was found to have a significant effect on the ASR expansion [12]. Studies show that the ASR expansion increase with increasing glass aggregate content, however, it is highly related to the fineness of glass aggregate [13,14]. Study shows that ASR expansion of glass mortars decrease with increasing glass sand finesses [15]. ASR is a surface-area dependent phenomenon, therefore it can be expected that ASR expansion will increase monotonically with fineness. In fact, there is a size of the aggregate at which the maximum expansion occurs (so-called “pessimum” size) [12]. Studies associated with the incorporation of fine waste glass aggregate has shown that optimum particle (aggregate) size that does not result in harmful expansions related to ASR is less than approx. 1–1.18 mm [1,16]. In that case brown and green soda-lime glass aggregate can be incorporated as replacement for natural sands at level of 100%. Studies show that green and brown glass sand mortars proved to be innocuous, regardless the replacement level, whereas clear glass can exhibit potential deleterious properties [12,15]. Equivalent observation was reported by Ling and Poon [11] who indicated that all cement mortars containing soda lime-glass met the permissible limits at satisfactory level. 

Recent studies show that cementitious composites containing up to 100% replacement ratio of brown-colored soda-lime fine aggregate with aggregate size less than 2.36 mm [11] and 4.75 mm [17] can be successfully incorporated into concrete without large ASR expansion. However, higher glass content (above 50%) can lead to the significant increase of water absorption in cementitious composites [7,10].

To suppress ASR effect on waste glass aggregate, various supplementary cementitious materials (SCMs) such as fly ash, ground blast furnace slag, metakaolin, silica fume, and nanosilica are being successfully incorporated [15]. In addition, reports show that fine waste glass powder greatly reduce the ASR expansion induced by glass aggregate [13,15]. Studies conducted by Aly et al. [18] have shown that glass powder hinders the expansion in comparison to the control specimen. Moreover, hybrid incorporation of waste glass and nanosilica greatly reduced the possible ASR [18].

Despite some potential inconveniences associated with application of waste glass, there are also few positive aspects of utilization the waste glass in cement-based materials. Application of waste glass can improve the resistance to an elevated temperature of cement-based composites [4]. Moreover, studies show that due to impermeable and smooth surface of the glass cullets, the use of waste glasses as an aggregate can improve the workability of the cementitious composite [4,14,17], however this findings are still disputative [17].

In the last decade, use of nanomaterials to produce cement-based composites has become a very important issue. The highest interest in industrial application of nanomaterials governs titanium dioxide (TiO_2_) and nanosilica (NS).

Nano-TiO_2_ is low cost material exhibiting photocatalytic, self-cleaning and antifogging properties [19,20,21,22]. Due to its unique properties TiO_2_ is widely applied to produce coatings on glass substrates with antireflective and self-cleaning properties [23,24,25]. Moreover, TiO_2_ nanoparticles find its utilization in building materials applied for façades such as coating, paints, mortars, plasters or concretes. Portland cement modified with nano-TiO_2_ particles has already been successfully applied in facades exposed to highly polluted environment [19,20,26]. In addition, there is a great amount of research confirming its potential application in self-cleaning cement mortars and concretes [26,27,28,29].

Incorporation of nanosilica (as a powder, dispersion or colloidal silica sol) is mostly aimed to improve the mechanical properties of cement-based composites. Due to its high surface area and chemical activity, nanosilica can contribute to significantly improve mechanical properties of cementitious composites, even when small amounts of nanomaterial are applied [30,31,32,33,34]. High surface area to volume ratio, and high water demand of nanoparticles associated with this phenomenon can contribute to significant decrement of workability. Therefore, various methods of nanosilica incorporation are proposed by researchers to overcome this obstacle. Nevertheless, incorporation route might affect final properties of cementitious composite [34].

The quality of people’s lives in big agglomerations is seriously impaired by factors such as air pollution from vehicle exhausts, and diseases caused by pathogenic microorganisms. Gram-negative coliforms such as *Escherichia coli* are common in domestic areas, for many strains are part of the human and animal microbiomes [35]. Some strains such as *E. coli* O157:H7 are pathogenic and can cause serious health problems. These Gram-negative bacteria are responsible for numerous nosocomial infections and food spoilages [36,37]. Consequently, there is a growing call for the methods to develop photocatalytic mortars that absorb pollutants or harmful substances, reduce (preferably eradicate) microorganisms on the surface, and contribute to a more sustainable construction [38]. High level of pollution, often occurring in human agglomerations, creates a necessity to develop novel, environmentally friendly strategies and approaches to decrease the pollution rate in urban centers. Incorporation of nanomaterials within cementitious composites and utilization of building surfaces as a cleaning media seems to be a key to improve human life and epidemiological safety, due to their versatile applicability [26]. The conception of similar materials was shown by Guo et al. [39]. With their nano-TiO_2_-based mortar, the authors were able to heighten NO removal and the reduction in number of bacterial cells in comparison to control material. 

Despite high amount of papers related to the influence of waste glass on the properties of cementitious composites only few are devoted to the practical production of such composites. Moreover, most of the studies aim to replace sand with waste glass in small amounts (e.g., 20%). In terms of practical application, replacement of sand with fine recycled glass aggregates up to 100% in cement-based composites could be a valuable alternative and support for sustainable development in the building sector and simply the production process. Although colorless waste glasses have been recycled effectively, colored waste glasses (especially brown soda-lime glass) in spite of their low recycling rate has gathered attention as a potential aggregate in cement-based composites [3]. This type of glass is commonly used for beverage containers, thus high amount of WG is consequently dumped in landfills. Therefore, there is a strong necessity to utilize this particular type of waste glass [11,17,40].

In addition, effect of nanosilica on the properties of cement mortars containing waste glass seems to be understudied. The aim of this study is to evaluate the possibility of manufacturing sustainable and self-cleaning cement mortars with use of commercially available nanomaterials and maximum possible brown soda-lime waste glass content. This study may positively contribute to the knowledge regarding practical application of nanomaterials and recycling materials to develop novel, sustainable, self-cleaning, and cement-based composites of bactericidal properties.

## 2. Materials and Methods

### 2.1. Materials

Cement CEM II/A-S 42.5 R (according to EN 197-1 standard) [41] containing nanocrystalline titanium dioxide has been used. The chemical composition of cement is presented in Table 1. Based on X-ray fluorescence analysis (XRF) presented in the table commercially available cement contained 2.72% of titanium dioxide (TiO_2_).

**Figure 1:**
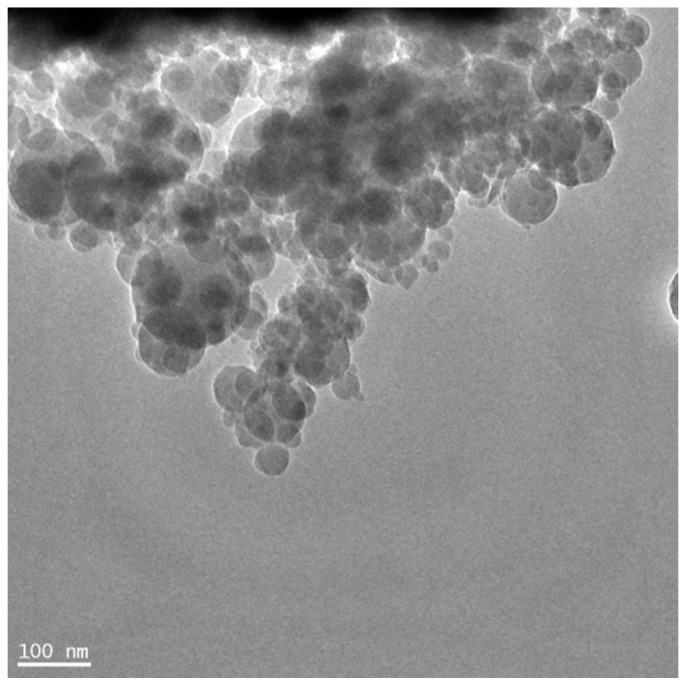
TEM micrograph of amorphous nanosilica (NS).

Amorphous nanosilica (NS) suspension containing 20 wt% of solid material was used. In Figure 1 the morphology of NS under the transmission electron microscope (TEM) is presented.

**Figure 2:**
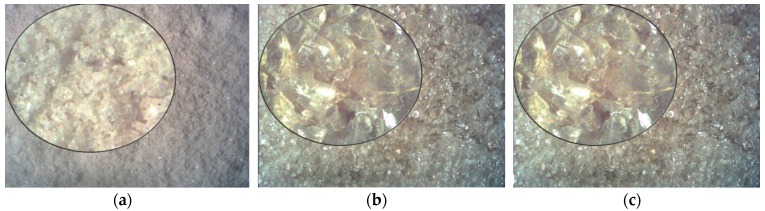
Shape and surface texture of different fine glass fractions: (**a**) 0.16–0.50 mm; (**b**) 0.50–1.0 mm; (**c**) 1–2 mm.

Quartz fine aggregate conforming EN 196-1 [42] was used for this study. Brown soda-lime waste glass (WG), widely used for bottles, was obtained from a local recycling company and applied as quartz sand replacement (Figure 2). Choice of brown soda-lime glass was based on fact that this glass is stored separately. In addition, mixed colored glass can exhibit different chemical composition (due to different origin) and impurities, which can highly affect the properties of cement mortar.

**Figure 3:**
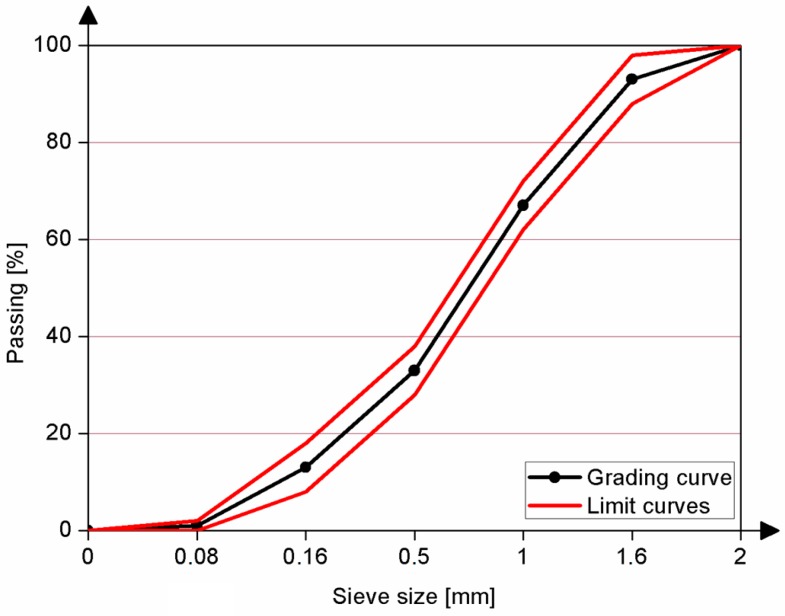
Particle size distribution of aggregate.

Waste glass was washed with water in order to remove organic contaminants, dried, and crushed in the mill to five different sizes. Identical grading was conducted for both the WG and quartz aggregates in order to compare the results and is presented in Figure 3 and Table 2.

Bacteria used in this study were a reference Escherichia coli K-12 (C600 derivate), from the Collection of the Department of Immunology, Microbiology and Physiological Chemistry at West Pomeranian University of Technology, Szczecin, Poland. The experimental strain was kept in −20 °C in Tryptone Soy Broth medium (TSB; Oxoid) supplemented with glycerol 10% (v/v). Before the tests bacteria were revived and pre-incubated on Tryptone Soy agar medium (TSA; Oxoid) for 24 h in 37 °C.

### 2.2. Cement Mortar Composition

Cement mortar components were mixed according to EN 196-1 standard [30] with the water to cement (w/c) ratio equal to 0.5. In samples containing nanosilica addition (3 wt%), the suspension was stirred with the mixing water (tap water) at high speed for one minute in order to ensure uniform dispersion of nanomaterial. Consistency of fresh mortars has been determined by flow table method according to EN 1015-3 standard [43]. Fresh mortar was poured into oiled molds to form samples with a size of 40 mm × 40 mm × 160 mm in accordance with the requirements of EN 196-1 [30]. The samples were demolded after 24 h and then cured for two, 7 and 28 days in a standard water bath at a temperature of 20 °C ± 2 °C. After the selected days of curing, flexural and compressive strength of samples has been determined. Proportions of all mixture components were given in Table 3. Moreover, samples containing 0% and 100% of waste glass were selected for bactericidal and mercury intrusion porosimetry (MIP) tests in order to determine the influence of waste glass and nanosilica on these properties. 

The MIP method is a common method used to characterize the pore structure in porous materials due to its simplicity, quickness, and wide measuring range of the pore diameter [44,45]. In order to obtain the pore size distribution of cement mortars, MIP test was performed on small-cored samples taken out of the specimens. After 28 days of curing, samples were transferred to a freeze-dryer in order to stop the hydration and to remove moisture of the pores. The surface tension of mercury was set at 0.48 N/m, whereas the contact angle for the intrusion at 140°. Samples were primarily tested at low pressure (up to 0.34 MPa). Afterwards, the cells with samples filled with mercury were weighed and placed in a pressure chamber where they were exposed to high pressures (up to 413 MPa).

### 2.3. Viability of Bacterial Cells after Contact with the Surface of the Mortar

The reduction in quantity of bacterial cells was performed using the spread plate test. The procedure of the antimicrobial activity was performed similarly to method presented by Zhu et al. [46] with minor modifications. The test was performed on sterilized cement mortars irradiated with the UVA light for 60 min. After irradiation, 25 µL of overnight culture were poured onto the surface (2 cm × 2 cm) of the mortar and incubated for 0, 15, and 30 min. After this time mortars were transferred into sterile falcon tubes (50 mL) and 10 mL of Maximum Recovery Diluent (MRD; Sharlau) was added. Then, tubes were vortex for 1 min and mortars were removed aseptically and imprinted on sterile TSA medium in order to control the adherence to the surface. In the next step the suspensions were prepared using serial dilution method and 100 µL·s^−1^ from each tube was transferred to sterile solid media (TSA) on Petri dish. Every sample was prepared in triplicate. Plates were incubated for 24 h in 37 °C. Afterwards, the grown colonies were counted and results were noted in logarithmic scale, here indicating the number of colony forming units per milliliter (CFU × mL^−1^). Statistical measures such as median and standard deviation were also used in the verification of the results.

## 3. Results and Discussion

### 3.1. Consistency

**Figure 4:**
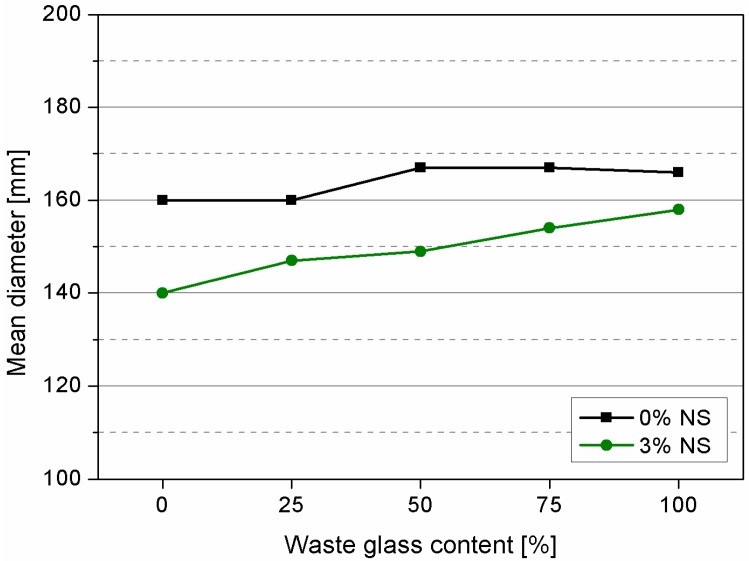
Consistency of tested mortars determined by flow table method.

Consistency of fresh mortars has been determined by flow table method and results are depicted in Figure 4. As it can be noticed the increase of waste glass replacement could slightly improve the fluidity of fresh mortar. When nanosilica was applied, decrement of consistency was observed. Moreover, with the increase of waste glass content, the consistency was also increasing and the final specimen containing 100% of waste glass and nanosilica (G100NS) exhibited basically the same consistency as reference sample containing quartz sand (R).

Decrement of consistency in presence of NS was attributed to high surface area to volume ratio of NS, which results in high water demand. Hence, significant reduction in fluidity and creation of local agglomerates of non-dispersed nanomaterial could be observed. In addition, high amount of nanomaterials reduced the content of free water for hydration process, and caused problems in achieving desired workability. Results of consistency determination confirmed findings of other researchers that waste glass due to its lower specific surface and smooth impermeable surfaces usually reduces the water demand. Therefore, there were no observed problems with incorporation of nanosilica.

### 3.2. Flexural Strength

**Figure 5:**
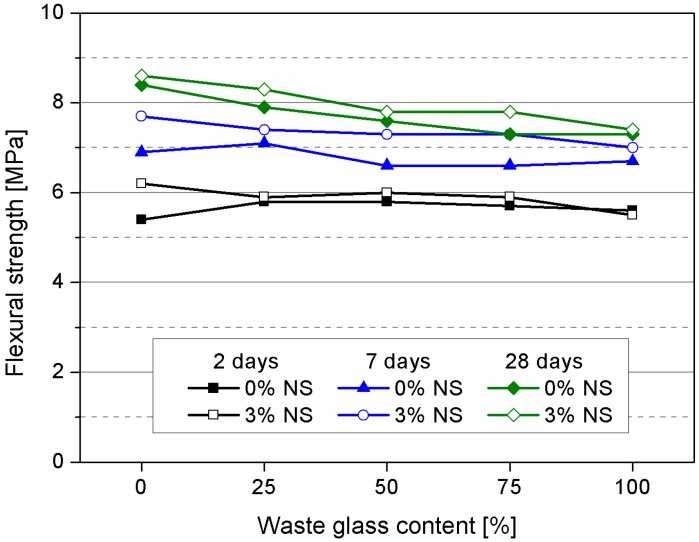
Flexural strength of tested mortars after 2, 7 and 28 days of curing.

Results of flexural strength after two, 7 and 28 days of curing were given in Table 4 and Figure 5. After two days of curing samples containing nanosilica already exhibited slight improvement in flexural strength and this can be attributed to properties of nanosilica. After 7 days of curing the effect of nanosilica can be observed more significantly. Strength improvement is attributed to the pozzolanic reaction and nano-filling effect of NS.

In general with the increase of glass content the flexural strength has been reduced, but due to presence of nanosilica sample containing 100% waste glass (S100NS) exhibited same strength as the control sample R. Results of 28 days flexural strength showed similar relation. The effect of nanosilica presence is noticeable, although, increment of glass content contributes to flexural strength loss.

### 3.3. Compressive Strength

**Figure 6:**
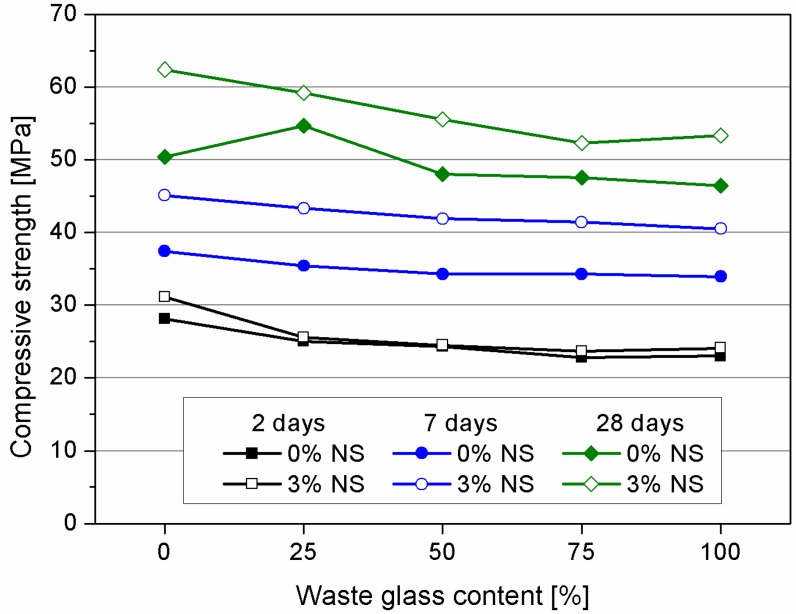
Compressive strength of tested mortars after 2, 7 and 28 days of curing.

The positive effect of nanosilica in cement mortars containing waste glass is more noticeable in the compressive strength development. Results of compressive strength determination are presented in Table 4 and depicted in Figure 6. It can be noticed from the first days of curing that samples containing higher content of WG have tendency to decrease their strength. Application of nanosilica after 2 days did not affect significantly the strength of mortars. Conversely, the presence of nanosilica in samples after 7 days of curing is significant when compared with the two-day strength sample. Likewise, with the increase of waste glass content the strength is decreasing. However, every sample containing nanosilica had higher compressive strength in comparison with plain reference sample containing quartz sand (R). 28 days compressive strength test confirms previous studies held by researchers that application of waste glass in quantities around 10%–30% improves the compressive strength, and beyond this amount the compressive strength has tendency to decrease. This effect of improvement when 25% of WG is applied is not noticeable in presence of nanosilica in the mix. The probable reason for such observation might be attributed to very beneficial influence of NS on the mechanical properties of cement mortars containing quartz aggregate (difference between R and RNS) where the bond between aggregate and cement matrix is already satisfactory (while compared to samples containing waste glass). Hence, nanosilica can be utilized entirely to further improvement microstructure. 

In general, all tested samples containing waste glass and NS had higher compressive strength than the reference sample. What is worth noting sample containing 100% of waste glass of nanosilica (G100NS) had still higher compressive strength than reference sample containing quartz sand (R). However, as mentioned the highest impact of nanosilica presence is noticeable in samples containing quartz sand.

**Figure 7:**
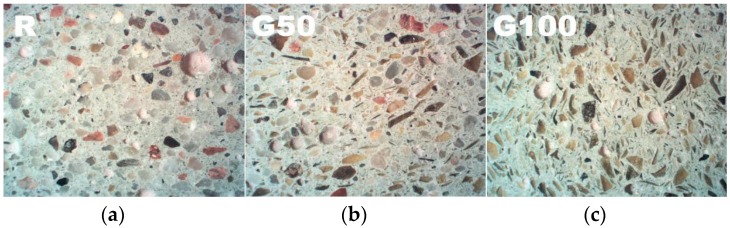
Microstructure of cement mortars containing different waste glass content. (**a**) 0%; (**b**) 50%; (**c**) 100%.

As it can be noticed from the presented results, the strength deterioration is highly related with glass content in the mix. In higher mix proportions, the addition of waste glass aggregate was found to negatively affect the mechanical properties of cement mortars. The reduction in the strength is caused mainly due to grain shape and its smoothness. Figure 7 presents the structure of mortars containing different glass content. There is a visible difference in the shape of waste glass and quartz aggregate. Milled waste glass aggregate is flat, needle-shaped and elongated. Therefore, we assume that high content of waste glass aggregate can lead to high stress concentrations, thus a reduction in strength. Moreover, due to the poor geometry of recycled glass it has been difficult to achieve a homogeneous distribution of aggregates, which is in accordance with results showed by Zheng [47].

As an effect of low water absorption of waste glass [48], the presence of WG aggregate particles led to a relative rise in w/c ratio, i.e. the efficient water content in the cementitious composite mix increased. Moreover, this could increase the flowing ability of fresh mortar (which was reflected in consistency test results). Study held by Limbachiya [48] has shown that due to very low water absorption and inherent smooth surface of WG aggregate, bleeding and segregation may occur [47]. In addition to increased water content in cement paste, smooth surfaces and sharp edges of glass particles resulted in an incomplete adhesion and weaker bond strength at the interfacial transition zone (ITZ) between glass particles and cement paste matrix comparing to rough natural sand aggregate [5,9,15,49]. Scanning electron microscope (SEM) analysis conducted by other researchers [49,50] revealed poor contact between the cement matrix and the recycled glass being a partial replacement of fine aggregate. In addition, poor homogeneity in the cement matrix probably inflicted by the smooth surface texture of the recycled glass waste, as well as inherent cracks in recycled glass particles likely caused by the crushing process (that can be considered as additional source of weakness) were observed by Zheng [47]. Moreover, increased percentage of recycled waste glass revealed more voids and cracks in the cement matrix [50].

Nevertheless, small quantities of replacement, due to the surface texture and strength of the glass particles (while compared to natural sand), improved the strength, which was noticed for mortar containing 25% (G25) waste glass. 

Presence of nanosilica significantly improved the compressive strength of cement mortars by compacting the structure and improving the bond between aggregate and cement matrix. These positive properties of NS can be mainly attributed to the size and large surface area, which has pozzolanic and filler effects on the cementitious matrix [51].

### 3.4. Water Absorption and Porosity

**Figure 8:**
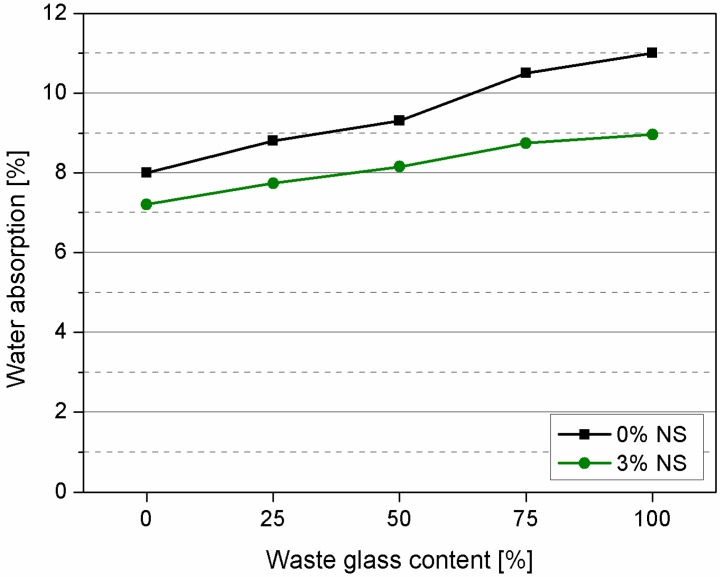
Water absorption of cement mortars.

Influence of waste glass and nanosilica on the microstructure of cement mortars was also determined by water absorption test which results are depicted in Figure 8.

Results show that waste glass content has a high impact on water absorption of cement mortars, especially when more than 50% of waste glass is used. Application of nanosilica helped to significantly reduce water absorption of cement mortars. In case of quartz sand (samples R and RNS) the influence of nanosilica is not so significant but the positive influence of nanosilica is highly noticeable with the increased content of waste glass (samples G75NS and G100NS). To support the findings hardened mortar (matrix) of the concrete samples were analyzed using mercury intrusion porosimetry (MIP).

**Figure 9:**
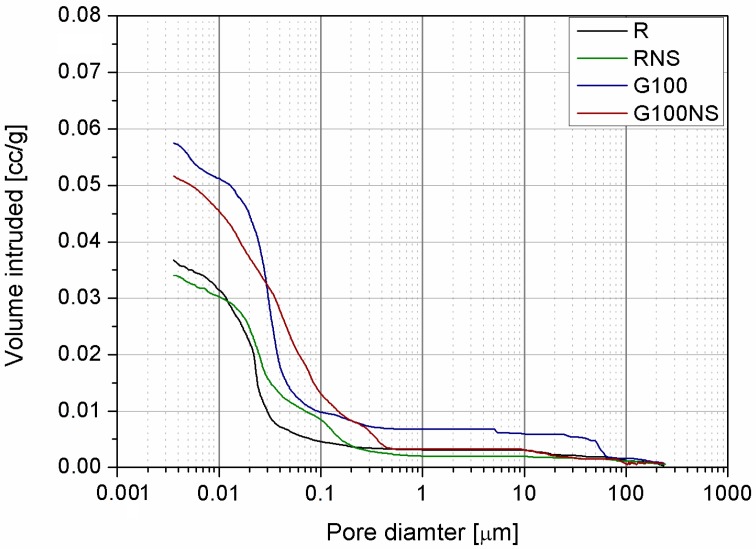
Mercury intrusion porosimetry results.

Results of MIP test are presented in Figure 9. It is clearly shown that the presence of waste glass highly increase total porosity of cement mortars which can be translated to higher water absorption. In order to gain more insight into the pore size distribution of samples, the measured values are divided into three size ranges: mesopores (5–50 nm), middle capillary pores (50–100 nm) and larger capillary pores (>100 nm) [52,53,54]. It should be noted that samples containing waste glass contained high amount of large capillary pores. It was caused by poorer cohesion between the glass aggregates and cement paste, which was an effect of smooth impermeable surfaces of WG aggregate. As stated by Neville [55] this critical interval (middle and large capillary pores) is mostly responsible for permeability and penetration of harmful substances into concrete.

Smooth surface of WG aggregate and relative increase in water to cement ratio (as a result of low water absorption of aggregate) contributed to the increased content of higher capillary pores [55]. In addition, as results of increased w/c, pore size distribution curve of mortars containing waste glass resulted in shift to the larger pores region. This observation is consistent with results gained by other researchers [45,52,54,56].

While nanosilica was incorporated to cement mortar, the pore structure has been altered. Firstly, the incorporation of nanosilica into the mixtures led to a decrease in total porosity. Moreover, it can be seen that MIP plots of mixtures containing nanosilica stand below the reference mixes, particularly in the range 0.2–100 µm. Presence of nanosilica made the pore structure of paste more homogeneous by increasing porosity in medium capillary range. Quercia et al. [57] observed that in presence of NS total pore volume is not necessarily changed, but larger pores subdivide into smaller pores. In the presented paper both, decrease of total porosity and alteration of pore structure have been shown. An equivalent observation has been reported by Oltulu and Sahin [58], where the addition of nanosilica to mortar dramatically changed the pore distribution, due to the proven behavior known as nanofiller effect of nanosilica. Even though porosity of samples remained similar or slightly reduced, the pore specific surface area became greatly affected while nanosilica was present in the mix. 

The obtained results of water absorption and MIP test show that application of nanosilica is effective in reducing the water absorption and refining the pore structure. These results are in accordance with findings presented by Ghafari et al. [51].

### 3.5. Bactericidal Properties

**Figure 10:**
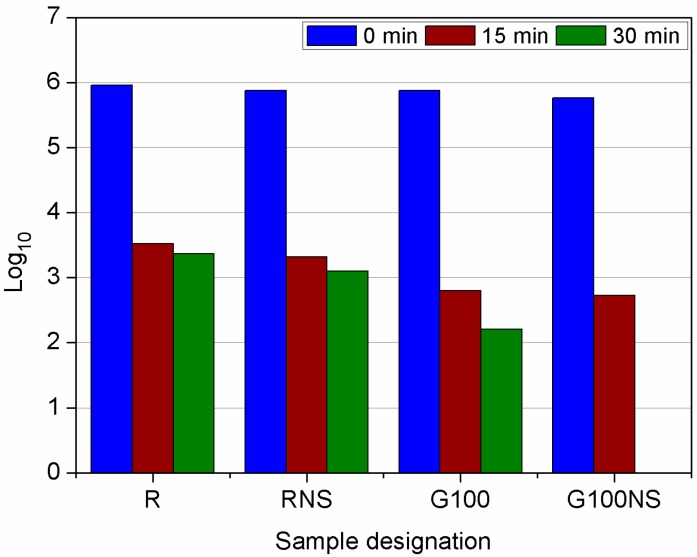
Reduction in quantity of viable bacterial cells (in logarithmic scale; CFU × mL^−1^) after contact with the cement mortars irradiated with UV-light for 0, 15 and 30 min. The experiment was conducted in triplicate (representative results are shown).

Results of bactericidal tests are presented in Figure 10. The outcome confirmed that the type of used mortar had had significant importance. The photocatalytic activity of mortar was improved when waste glass was incorporated as an aggregate. The incorporated waste glass aggregate might enable the TiO_2_ within the inner part of the surface layer to play role in a bactericidal reaction. As reported by Chen et al. [59] and van Lieshout et al. [60] the light transmittance property of waste glass play a vital role in the efficiency enhancement of photocatalytic reactions. This is believed to be related to the high light transmitting characteristic of the recycled glass particles. Therefore, light could be carried to a greater depth activating the TiO_2_ on the surface as well as within the surface layer [26]. However, in presented work this effect could not play a main role in affecting the bactericidal properties due to the fact that brown soda-lime glass is characterized in relative low light transmittance [59]. For that reason, we assume the bactericidal effect to be rather attributed to the high porosity of structure, which is believed, can be favorable to the internal dispersion of pollutants beneficial for pollutant and bacteria removal [61]. As it is stated by Chen et al. [59] porous structures, which have a larger surface area are beneficial for the adsorption process. Therefore, a high number of pores together with high total surface area can be favorable to the internal dispersion of pollutants and bacteria removal. This property has been reflected in the slight reduction in quantity of viable bacterial cells of samples containing nanosilica (RNS and G100NS). Larger pores subdivided into smaller pores that greatly increased specific pore surface area [58]. However, on one hand higher porosity facilitates the adhesion and survival of micro-organisms and on the other, a higher porosity provides larger surface area, which is favorable for biocidal contact surface [35].

**Figure 11:**
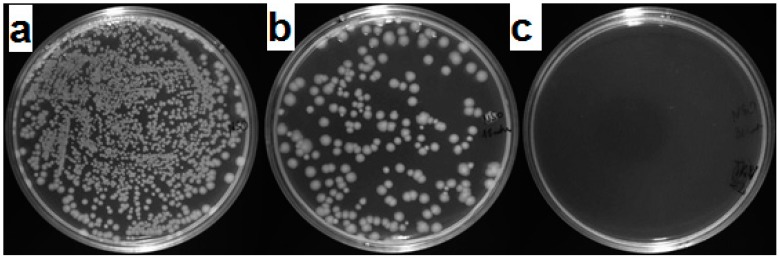
Reduction in quantity of viable bacteria in time on TSA medium after contact with G100NS (cement mortar); (**a**) 0 min, (**b**) 15 min; (**c**) 30 min.

Although the conditions used for the test were established for the maximal preservation of bacteria, time of inactivation was relatively short. Verdier et al. [62] indicated in their studies that time required to eliminate bacteria from the surface of cement mortar is 2–4 h, albeit without the use of the UV light. However, they confirmed that this factor is likely to shorten time of inactivation. This was also accurate in other studies in which time required to decrease the number of cells by 75%–99% was ranging from 30–90 min [63]. In our experiments the reduction in quantity of bacteria was statistically significant in comparison to the control after 15 min of contact with the mortars, although higher reduction (complete for the G100NS mortars) was noticed in samples containing waste glass. Statistical differences between plates occurred after 30 min of treatment, which is shown in Figure 11. The highest rate of inactivation was observed for mortar containing 100% of waste glass and nanosilica (G100NS). The mortars used in this study were irradiated UVA light that is relatively weak and present in the sunlight. Therefore, we assume that proposed mortars may show bactericidal activity exposed to the natural daylight.

Control imprints on agar plates (TSA) did not show signs of effective adherence of the bacteria to the surface, which has additionally confirmed previously known feature of this bacteria that it shows poor adhesion abilities. Obtained results were repeatable.

In general, the mortars containing waste glass were characterized by a higher value of the total open porosity with respect to the reference mortars, therefore the effect of bacteria inactivation was more significant. What is worth noting is the fact that presence of nanosilica altered bactericidal properties of cement mortars. This effect of nanosilica can probably be attributed (as mentioned before) to its influence on the pore structure. It can be noticed from MIP analysis that nanosilica refined the pore structure by reducing the pores in large capillary area and increasing in middle range. Likewise, the total porosity of cement mortars was slightly reduced; however, due to refinement of pore structure, more surface area was provided for bacteria inactivation process, compared to the samples without addition of nanosilica. This effect was much more visible for sample containing waste glass due to significantly higher total porosity of mortars. Despite positive results, the effect of pore size distribution and total porosity in presence of nanosilica on bactericidal properties requires further investigation.

## 4. Conclusions

The experimental study herein described was conducted aiming to evaluate the effect of brown soda-lime waste glass on the mechanical and bactericidal properties of cement mortars. Application of waste glass as a fine aggregate to cement mortars seems to be interesting approach to produce sustainable self-cleaning composites, however, few inconveniences must be taken into account. On the basis of the results presented in this work, following conclusions have been drawn:

- Due to smooth impermeable surfaces of WG aggregate, the consistency of cement mortars is increased while compared to mortars containing quartz aggregate. However, poorer cohesion between the glass aggregates and cement paste (as a result of smoothness and grain shape of WG) contributes to flexural and compressive strength decrement.
- Presence of waste glass highly affects the microstructure of cement mortars by increasing the total porosity and water absorption of mortars, although this might cause higher efficiency in the inactivation of bacteria.
- Nanosilica due to its high pozzolanic activity and nanofiller effect improves the bond between the waste glass and the cement paste resulting in improving the final properties of cement mortars. In addition, nanosilica improves the microstructure of cement mortars by reducing the total porosity, refining the pore structure a decreasing water absorption of mortars. It was observed that sand can be successfully replaced with 100% of brown-soda waste glass (to obtain similar mechanical properties) while optimum amount of nanosilica is incorporated.
- Incorporation of waste glass as an aggregate in cement mortars containing cement modified with nanocrystalline titanium dioxide can improve the bactericidal properties of cement mortars against Gram-negative coliforms. Moreover, presence of nanosilica contributes to the pore structure refinement that increases the specific pore surface area, which is favorable for bacteria removal.
- Studied bacteria were inactivated in relatively short time (only 30 min), which is an advantage of the proposed mortar G100NS, because the shorter inactivation time is, the lower is the risk of spreading of bacteria in the environment.

## Figures and Tables

**Table 1:** Chemical composition of CEM II/A-S 42.5 R containing titanium dioxide.

TiO_2_	SiO_2_	Al_2_O_3_	Fe_2_O_3_	CaO	MgO	SO_3_	Na_2_O	K_2_O	Cl^−^	LOI
2.72	17.2	5.29	1.00	36.25	1.57	0.00	0.00	0.47	0.00	3.5

**Table 2:** Grading of sand and waste glass (WG) aggregates.

Sieve Size (mm)		Mass (%)
Passing	Retained	
2.00	1.60	7 ± 5
1.60	1.00	33 ± 5
1.00	0.50	67 ± 5
0.50	0.16	87 ± 5
0.16	0.08	99 ± 1

**Table 3:** Mixture proportions of mortars, kg/m^3^.

Sample Designation	Unit Weight (kg/m^3^)				Sand Replacement with WG (%)
	Cem II/A-S 42.5 R with Nano-TiO_2_	Water	Nanosilica Suspension	Sand	
R	519	257	-	1546	0
G25	519	257	-	1159	25
G50	519	257	-	773	50
G75	519	257	-	773	75
G100	519	257	-	-	100
RNS	519	195	78	1546	0
G25NS	519	195	78	1159	25
G50NS	519	195	78	773	50
G75NS	519	195	78	386	75
G100NS	519	195	78	-	100

**Table 4:** Flexural and compressive strength after 2, 7 and 28 days of curing.

Sample Designation	Flexural Strength (MPa)			Compressive Strength (MPa)		
	2 Days	7 Days	28 Days	2 Days	7 Days	28 Days
R	5.4	6.9	8.4	28.1	37.4	50.4
G25	5.8	7.1	7.9	25	35.4	54.7
G50	5.8	6.6	7.6	24.3	34.3	48
G75	5.7	6.6	7.3	22.8	34.3	47.5
G100	5.6	6.7	7.3	23	33.9	46.4
RNS	6.2	7.7	8.6	31.1	45.1	62.4
G25NS	5.9	7.4	8.3	25.6	43.3	59.2
G50NS	6.0	7.3	7.8	24.5	41.9	55.5
G75NS	5.9	7.3	7.8	23.7	41.4	52.3
G100NS	5.5	7.0	7.4	24.1	40.5	53.3
